# Effects of Soybean Agglutinin on Mechanical Barrier Function and Tight Junction Protein Expression in Intestinal Epithelial Cells from Piglets

**DOI:** 10.3390/ijms141121689

**Published:** 2013-11-01

**Authors:** Li Pan, Guixin Qin, Yuan Zhao, Jun Wang, Feifei Liu, Dongsheng Che

**Affiliations:** 1 College of Animal Science and Technology, Jilin Agricultural University, Changchun 130118, China; E-Mails: panli0628@126.com (L.P.); qgx@jlau.edu.cn (G.Q.); feikathy@163.com (F.L.); 2 Key Laboratory of Animal Production, Product Quality and Security, Ministry of Education, Jilin Agricultural University, Changchun 130118, China; E-Mails: zhaoyuan4CL52@126.com (Y.Z.); wangjun4169@126.com (J.W.)

**Keywords:** intestinal epithelial cell, mechanical barrier function, piglet, soybean agglutinin, tight junction protein

## Abstract

In this study, we sought to investigate the role of soybean agglutinin (SBA) in mediating membrane permeability and the mechanical barrier function of intestinal epithelial cells. The IPEC-J2 cells were cultured and treated with 0, 0.5, 1.0, 1.5, 2.0, 2.5, or 3.0 mg/mL SBA. Transepithelial electrical resistance (TEER) and alkaline phosphatase (AP) activity were measured to evaluate membrane permeability. The results showed a significant decrease in TEER values (*p* < 0.05) in a time- and dose-dependent manner, and a pronounced increase in AP activity (*p* < 0.05). Cell growth and cell morphology were used to evaluate the cell viability. A significant cell growth inhibition (*p* < 0.05) and alteration of morphology were observed when the concentration of SBA was increased. The results of western blotting showed that the expression levels of occludin and claudin-3 were decreased by 31% and 64% compared to those of the control, respectively (*p* < 0.05). In addition, immunofluorescence labeling indicated an obvious decrease in staining of these targets and changes in their localizations. In conclusion, SBA increased the membrane permeability, inhibited the cell viability and reduced the levels of tight junction proteins (occludin and claudin-3), leading to a decrease in mechanical barrier function in intestinal epithelial cells.

## 1. Introduction

Soybean agglutinin (SBA), accounting for 5%–7% of the anti-nutritional factors (ANFs) present in soybeans, is a major ANF. SBA mainly presents as dimers or tetramers, with multiple carbohydrate binding sites, which has the potential to bind to the receptor of cell membranes [1]. Indeed, SBA can bind to carbohydrates on the brush border membrane of intestinal epithelial cells that contain substantial amounts of *N*-acetylgalactosamine and *N*-acetylgalactose [2]. The binding of SBA can lead to toxic and anaphylactoid effects in animals, including decreased nutrient digestion and absorption, disruption of the bacterial balance, and damage to the the basic structure of intestinal mucosal cells [3].

In terms of immunity, the intestinal tract represents the first barrier of immune defense and is formed by intestinal epithelial cells. In recent decades, cell lines or primary cell cultures have been widely used to reveal cell proliferation and differentiation mechanisms in the intestinal tract [4]. These studies have demonstrated that selective barrier function of intestinal epithelial cells is maintained by intercellular contacts formed by tight junctions (TJs). TJs can limit the transport of relatively small molecules, regulate intestinal epithelial cell permeability, and protect mucosal cells from being attacked by bacteria and toxic macromolecules [5]. In addition, TJs regulate the maintenance of epithelial cell polarity [6].

TJs have a complicated protein composition including occludin and claudins [7–10]. Occludin is a 62–82 kDa protein first indentified as an important membrane component from chicken liver [11,12]. It performs specialized functions in maintaining cell polarity and para-cellular barrier functions. Abundance of occludin is related to the degree of sealing of epithelia [13]. Claudins are 21–28 kDa proteins chiefly involved in the function and structure of TJs [14,15], which participate in barrier function and fence function.

Disruption of cell-to-cell adhesions can be evaluated by measuring the integrity of TJs; reduced TJ integrity is the first indication of epithelial cell injury. Even the presence of undigested SBA in the intestinal tract can destroy the structure and function of the intestines [16]. However, few studies have investigated the concentration-dependent effects of SBA on the permeability of the intestinal epithelial cell barrier and the expression of TJ components.

In the present investigation, we sought to evaluate the effects of different concentrations of SBA on the barrier function of the intestine, TJ protein expression and distribution of intestinal epithelial cells in piglets at the molecular level. Our data provide an important theoretical basis for the discussion of SBA anti-nutritional mechanisms and for improving the value of the soybean protein.

## 2. Results and Discussion

### 2.1. Effects of SBA on Intestinal Permeability Barrier Function

#### 2.1.1. Trans-Epithelial Electrical Resistance (TEER) Value Analysis

**Figure 1:**
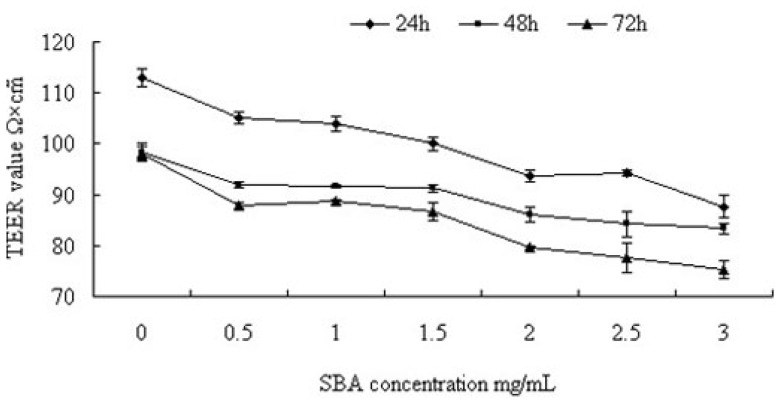
Effects of SBA on TEER in IPEC-J2 cells. Cells were treated with various concentration of SBA for 24 h, 48 h, or 72 h, TEER values are expressed in Ω × cm^2^ as the mean ± standard error from 3 independent experiments and presented a significant decline in a time and dose-dependent (*p* < 0.05). The control group was treated with 0 mg/mL SBA for the indicated times (24, 48, or 72 h). Treatment of 0.5, 1.0 or 1.5 mg/mL SBA group had a significant decrease compared with control (*p* < 0.05), in addition, 2.0, 2.5 or 3.0mg/mL had a significant decrease compared with 0.5,1.0 or 1.5 mg/mL SBA treatment groups (*p* < 0.05) (48 h or 72 h). However no significant differences were observed among 0.5, 1.0 and 1.5 mg/mL treatment, or between the 2.0 and 2.5 mg/mL SBA treatment (*p* > 0.05).

TEER was measured to analyze the effects of SBA on TJ integrity in intestinal epithelial cells. Interestingly, SBA treatment resulted in a time- and dose-dependent reduction of the TEER in IPEC-J2 cells (Figure 1). SBA (0.5 mg/mL) caused significant reductions in TEER values of 7.08%, 6.50%, and 10.4% when compared with the control (0 mg/mL SBA) at 24, 48, and 72 h, respectively (*p* < 0.05) and at this concentration, there were significant time-dependent decreases in the TEER values (*p* < 0.05). Similarly, significant differences were observed in cells treated with 0.5, 1.0, 2.0, or 3.0 mg/mL SBA treatment compared with control for 24 h (*p* < 0.05), and these treatments elicited significant dose-dependent decreases in the TEER values of approximately 7.08%, 4.9%, 17.20%, and 22.50%, respectively (a significant decrease among these groups. *p* < 0.05), compared with the control (*p* < 0.05). However, no significant decreases were observed between 1.0 and 1.5 mg/mL treatments groups; or 2.0 and 2.5 mg/mL treatments (*p* > 0.05). When the cells were treated with different concentrations of SBA for 48 or 72h, regular reductions were presented in TEER values (*p* < 0.05).

TEER is a typical indicator of epithelial integrity and permeability [5] and it was decreased by SBA in a time- and dose-dependent manner. These results were consistent with those observed in a study of wheat germ agglutinin [17,18]. The reduction in TEER was likely due to an effect on the plasma membrane, such as changes in transcellular ion transport pathways [19]. Moreover, specific binding of SBA with *N*-acetylgalactosamine and *N*-acetylglucosamine on the surface of intestinal epithelial cells alters the structure of the membrane, causing a loss of cytoplasm [20]. In addition to SBA, extractions from other dietary components, such as galangal, marigold, and hops were found to decrease the TEER in Caco-2 cells [21]. Our study showed that different concentrations of SBA (ranging from 0.5 to 3.0 mg/mL) had different effects on the TEER. This may be due to the varying degrees of interaction between the cells in the presence of various SBA concentrations [22]. Our data suggests that high concentrations and long reaction times had more significant effects; however, when the agglutinin concentration is raised to a certain degree, the treatment time no longer affects the result.

#### 2.1.2. Alkaline Phosphatase (AP) Activity Analysis

**Figure 2:**
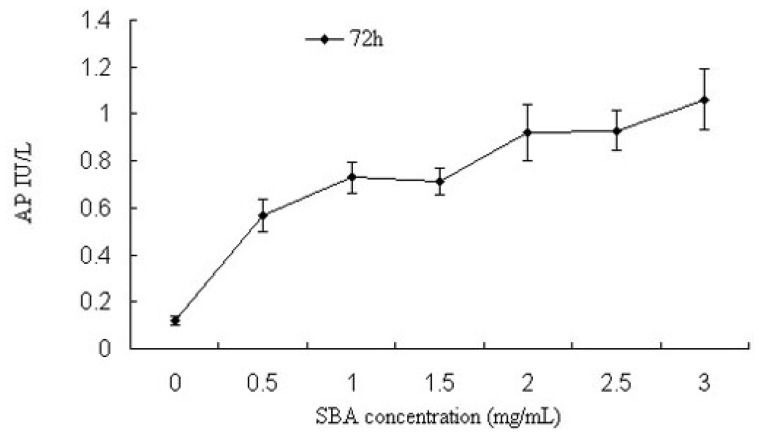
Effects of SBA on AP activity in IPEC-J2 cells. Cells were treated with various concentration of SBA for 72 h, culture supernatants were collected, and AP activity was measured. The control cells were treated with 0 mg/mL SBA for 72 h. Values are the mean ±SD from 4 independent experiments. Treatment of 0.5, 1.0 or 1.5 mg/mL SBA was significantly higher than control (*p* < 0.05), and the 2.0, 2.5 or 3.0 mg/mL SBA treatment were even higher than treatment of 0.5, 1.0 or 1.5 mg/mL (*p* < 0.05). However, no significant differences were observed among 0.5, 1.0 and 1.5 mg/mL treatment; or 2.0, 2.5 and 3.0 mg/mL SBA treatment (*p* > 0.05).

**Figure 3:**
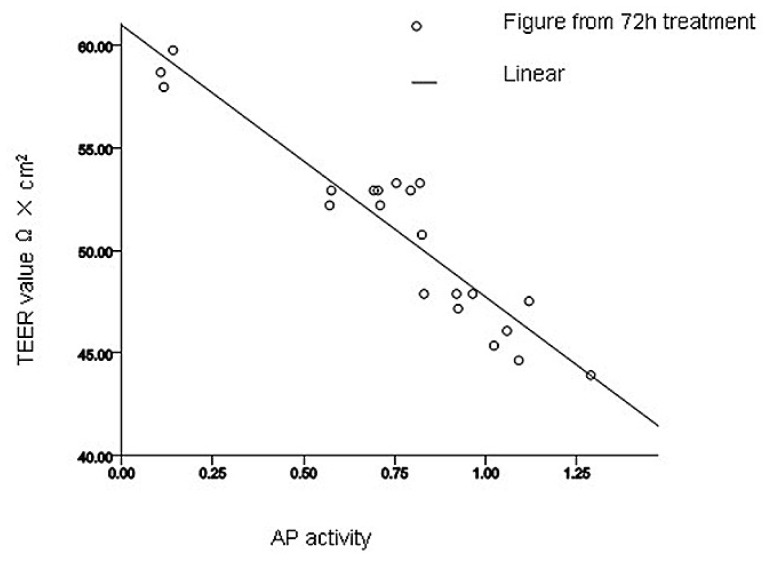
Linear correlations between AP activity and TEER value after 72 h treatment with 0, 0.5, 1.0, 1.5, 2.0, 2.5, or 3.0 mg/mL SBA. The formula was obtained by SPSS 17.0 software (SPSS Inc, Chicago, IL, USA. *n*_AP_ = 28, *n*_TEER_ = 21, *y* = 60.976 − 13.251*x*, *R*^2^ = 0.883, *R*^2^ = 0.883, *p* < 0.01, TEER values were dependent variable, AP activity were independent variable.

Alkaline phosphatase (AP) is a ubiquitous enzyme distributed among many tissues and cell types, whose activity is another indicator of cell membrane permeability. Next, we measured AP activity in cells after treatment with various concentrations of SBA for 72 h (Figure 2). With increasing concentrations of SBA, significant increases in AP activity were observed. Significant differences were observed in cells treated with 0.5 and 2.0 mg/mL SBA treatment (*p* < 0.05), exhibiting 3.6- and 6.6-fold increases in AP activity, respectively, compared to the control (*p* < 0.05), However, no significant differences were observed among 0.5, 1.0 and 1.5 mg/mL treatment; or 2.0, 2.5 and 3.0 mg/mL SBA treatment (*p* > 0.05). As observed in TEER and AP experiments, there was a linear correlation between the TEER value and AP activity after 72 h treatment (*p* < 0.05, Figure 3). Therefore, our data demonstrated that permeability of intestinal epithelial cells was sensitive to SBA.

Intestinal AP activity is highly expressed in intestinal tissue [23], and it has an essential function in maintaining epithelial integrity in intestinal cells. The loss of AP in these cells increases permeability, promoting inflammation and sepsis [24,25]. As shown previously [26], as the concentration of SBA increased, extracellular AP activity increased causing epithelial damage. Regression analysis revealed that AP had a linear relationship with TEER, suggesting that increased extracellular AP activity induced a reduction in the TEER, thus providing evidence that SBA has a vital influence on intestinal permeability.

### 2.2. Effects of SBA on Intestinal Epithelial Cell Viability and Cellular Morphology

#### 2.2.1. Cell Viability: MTT Assay Analysis

**Figure 4:**
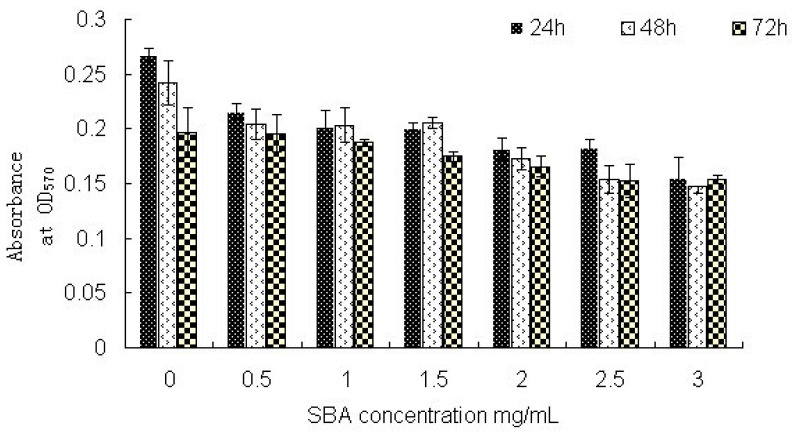
MTT assays showing the viability of IPEC-J2 cells after treatment with different concentrations of SBA for 24 h, 48 h, or 72 h. The data are the mean ± SD from 4 independent experiments and presented a significant decline trend (*p* < 0.05). The control group was treated with 0 mg/mL SBA for the indicated times (24, 48, or 72 h). Treatment with 0.5, 1.0 or 1.5 mg/mL SBA resulted in a significant decrease compared with control (*p* < 0.05), in addition, 2.0, 2.5 or 3.0mg/mL led to a significant decrease when compared with 0.5,1.0 or 1.5 mg/mL SBA treatment groups (*p* < 0.05) (48 h or 72 h). However no significant differences were observed among 0.5, 1.0 and 1.5 mg/mL treatment, or between the 2.0 and 2.5 mg/mL SBA treatment (*p* > 0.05).

3-(4,5)-dimethylthiahiazo(-z-y1)-3,5-di-phenytetrazoliumromide (MTT) was usually used as a yellow dye to detect cell survival and growth. Obvious reductions in absorbance (18.5%, 15.7%, and 0.5%, respectively) were obtained when cells were treated with 0.5 mg/mL SBA for 24, 48, or 72 h (*p* < 0.05) whereas no significant differences were observed when comparing the different time points (*p* > 0.05). Treatment with 0.5, 1.0, 2.0, or 3.0 mg/mL SBA for 24 h resulted in significant decreases in absorbance (18.5%, 23.8%, 31.3%, and 41.5%, respectively and a significant decrease among these groups. *p* < 0.05) compared to the control (*p* < 0.05, Figure 4). However, no significant differences were observed between 1.0 and 1.5mg/mL treatment; or 2.0 and 2.5 mg/mL SBA treatment (*p* > 0.05). When the cells were treated with different concentrations of SBA for 48 or 72 h, regular decreases in MTT values were found.

Cell growth is an indicator of the severity of SBA-induced inhibition of cell viability. SBA has been shown to accelerate lymph cell division activity [27]. The swallowed SBA in intestinal epithelial cells causes changes in the structure and function of the intestinal mucosa and affects crypt cell division [28]. However, a combination of abundant SBA and small intestinal mucosa epithelial cells causes intestinal villus atrophy and reduces the survival of epithelial cells in rats [29]. In addition, binding of SBA to the alimentary tract surface alters gastrointestinal cell morphology and metabolism, affecting cell proliferation [30]. In the present study, we found a significant decrease in cell viability after treatment with SBA, and the extent of reduced cell viability was time- and dose-dependent in response to SBA treatment. This experimental result was inconsistent with results reported by Liu (2011) [31], whose results showed a significant increase in cell viability on rabbit intestinal epithelial cell after treated with SBA for 24,48 or 72 h, most likely due to different reactions of SBA in different animal species [23]. Because of the different dietary structures of the animals used in our study and Liu’s study, differences in system evolution and individual developmental processes resulted in variations in the presence of sugar chains and the occurrence of glycosylation events on the gastrointestinal mucosa cellular surface. Thus, SBA would be expected to exert differential effects on the viability of intestinal epithelial cells and on membrane permeability in pigs versus rabbits.

#### 2.2.2. Morphometric Analysis

**Figure 5:**
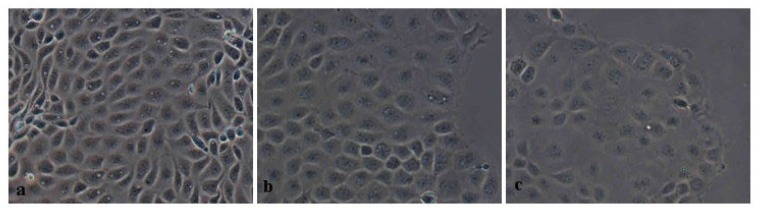
Representative microphotographs demonstrating the effects of SBA on the morphology and growth of IPEC-J2 cells. Cells were cultured to confluence and incubated with the 0 mg/mL (**a**), 0.5 mg/mL (**b**), 2.0 mg/mL (**c**) of SBA for 48 h; 0 mg/mL of SBA treated cells are shown as the control. Magnification, 200×.

Addition of SBA to IPEC-J2 cultures changed the morphology and growth of the cells (Figure 5). The overall area covered by the adhered cells indicated that treatment with 0.5 or 2.0 mg/mL SBA resulted in changes in cell morphology and state. With increasing concentrations of SBA, cell growth was significantly decreased and the boundaries between adjacent cells were ambiguous.

#### 2.2.3. Pearson Correlation Analysis

After incubation with different concentrations of SBA for 72 h, correlations between two of MTT values, TEER values and AP activity were obtained using SPSS software 17.0 (SPSS Inc, Chicago, IL, USA) (Table 1). MTT assay results and TEER values were significantly positively correlated, with a correlation coefficient of 0.688 (*p* < 0.01). AP activity had a negative correlation with both MTT assay results and TEER values, with correlation coefficients of −0.682 and −0.940, respectively (*p* < 0.01).

Another finding in this study was the vital correlation between cell viability and cell membrane permeability. The results of Pearson correlations demonstrated a significant positive co-relationship between TEER value and cell viability, and with increasing cell membrane permeability, inhibition of cell viability was clearly observed. A negative correlation between AP and cell growth was observed at the same time. AP is a major binding protein in the cell membrane and plays an important role in cell growth and metabolism [32]. AP participates indirectly in the transfer of calcium ions. As a major messenger, calcium ions are involved in the regulation of the physiological activities of cells and tissues, such as metabolism, cell division, and muscle contraction [33,34]. In our study, cell membrane integrity was reduced following stimulation with SBA, and extracellular AP activity was increased, causing an elevation of intracellular calcium ion concentrations, this may explain the inhibition of the cell proliferation and viability. However, further studies are required to investigate this mechanism of SBA-induced intestinal epithelial cells viability.

Together with previous data, these results indicated that different concentrations of SBA altered the integrity, permeability barrier function, and viability of intestinal epithelial cells in a time- and dose-dependent manner.

### 2.3. Effects of SBA on the Distribution and Expression of TJ Proteins

#### 2.3.1. Effects of SBA on the Distribution of TJ Proteins

**Figure 6:**
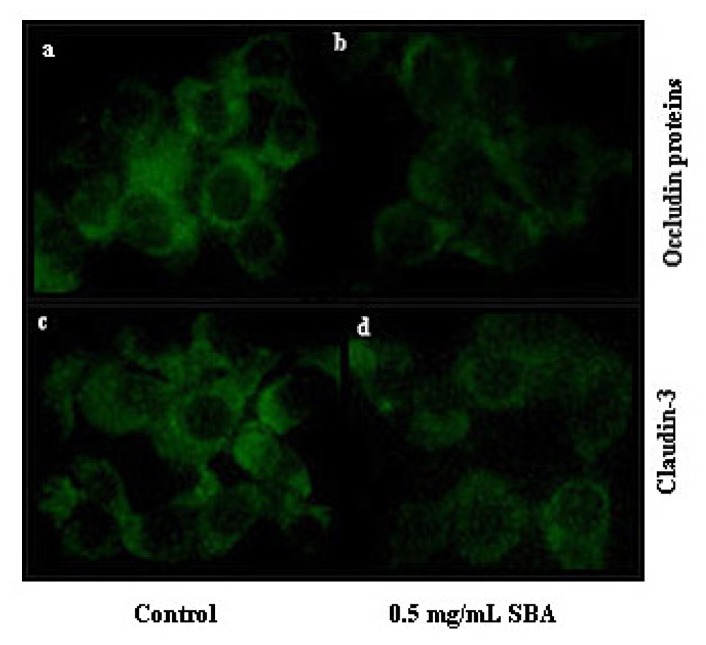
Effects of SBA on the distribution of tight junction proteins. The distributions of occludin treated with 0 mg/mL (**a**) or treated with 0.5 mg/mL (**b**) and claudin-3 treated with 0 mg/mL (**c**) or treated with 0.5 mg/mL (**d**), were determined by immunofluorescence. IPEC-J2 cells were grown on glass slides within 6-well plates until reaching complete confluence and were then treated with 0 or 0.5 mg/mL SBA for 48 h. The cells treated with 0 mg/mL SBA for 48 h were used as a control. Representative images are shown at a magnification of 400×.

TJs determine the integrity and permeability of epithelial cells. Therefore, we next tested the effects of SBA on the distribution and expression of TJ proteins (occludin and claudin-3) using immunofluorescence labeling and confocal microscopy. These TJ target proteins were located at the cell-cell contact regions, as shown in (Figure 6). However, after treatment with 0.5 mg/mL SBA, the morphology of the cells was altered and the staining intensity and membrane localization of occludin and claudin-3 proteins were obviously decreased when compared with the control.

#### 2.3.2. Effects of SBA on the Expression of TJ Proteins

**Figure 7:**
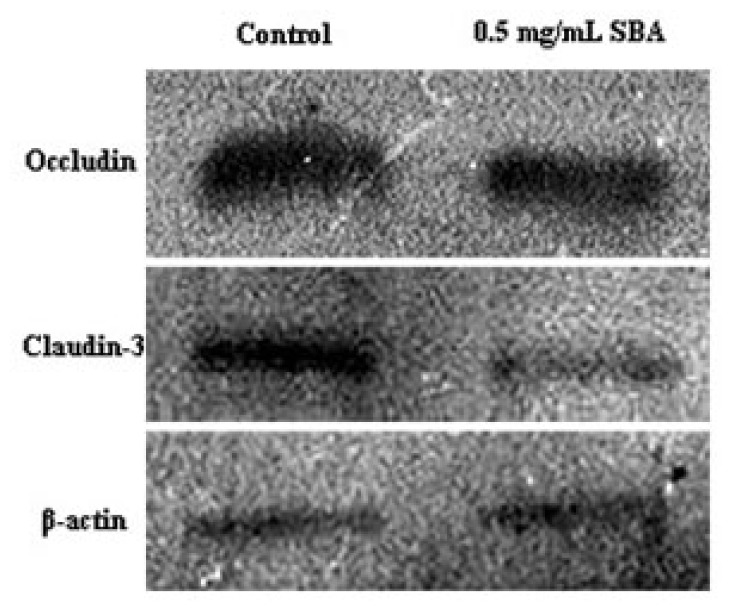
Western blotting of tight junction proteins following SBA treatment. Fifteen microliters of 0 (Control) or 0.5 mg/mL SBA-treated total protein extracts were analyzed by immunoblotting with antibodies for occludin and claudin-3. β-actin was used as a loading control. Representative western blots from 4 independent experiments are shown.

Western blot analysis revealed obvious decreased in the expression of occludin and claudin-3 after a 48-h treatment with 0.5 mg/mL SBA when compared with those of the control (Figure 7). Densitometric analysis of western blotting demonstrated that the signals for occludin and claudin-3 were reduced by 31% and 64%, respectively, following SBA treatment of IPEC-J2 cells (*p* < 0.05).

Daily intake of SBA increases the permeability of the intestinal wall, resulting in the loss of protein from the gastrointestinal tract [35]. Alteration of cellular permeability can be used to reveal changes in the TJ complex [36]. In the present study, we observed a significant reduction in the staining intensity and a reduction in the expression of occludin and claudin-3. Occludin and claudins proteins have been identified as TJ-associated proteins, that localize to the ridge line of the intermembrane of TJs [11]. Occludin has been linked to the regulation of intermembrane diffusion and paracellular diffusion of small molecules [37]. Moreover, occludin expression increases and it redistributes upon treatment of rat jejunum crypt epithelial cells (IEC6s) with gliadin [38]. Overexpression of mutant occludin led to the alteration of TJ barrier function [39,40]. Claudin proteins are regarded as the structural backbone of TJs [41] and seal the space between two adjacent cells. Addition of exogenous claudin proteins to fibroblasts, which lack endogenous TJ proteins, could promote the formation of TJ proteins [42], and increase the cellular barrier function. TJ junctions are associated with the actin cytoskeletion [43,44], which can regulate TJ structure and paracellular permeability [45]. SBA can also decrease the expression of ZO-1 in mid-jejunum tissue of piglets [46]. When injured or non-injured endothelia were exposed to SBA, significant alterations of cellular appearance and actin cytoskeleton distribution were observed [47]. This may explain the alteration of cellular morphology in Figures 5 and 6. As a consequence, treatment with SBA can decrease the expression of TJ components, thereby damaging the intestinal integrity and barrier function, as evaluated by TEER, AP activity, cell viability, and cellular morphology. These findings suggest that occludin and claudins have vital functions in maintaining epithelial integrity in response to SBA.

## 3. Experimental Section

This study was based on the culture of IPEC-J2 cells and was divided into two parts. The first was the evaluation of intestinal epithelial cell permeability and viability, and the second was the measurement of protein expression and distribution within TJs.

### 3.1. Cell Culture

IPEC-J2 cells were obtained from Professor Wu Guoyao of China agricultural university and were grown in cell culture flasks using Dulbecco’s modified Eagle medium (DMEM)/F12 medium (Gibco, Carlsbad, NM, USA) supplemented with 10% fetal bovine serum (FBS, Gibco, Carlsbad, NM, USA), 1% penicillin-streptomycin (Sigma, St. Louis, MO, USA) and 1% glutamine (Amersco, Solon, Tucson, AZ, USA) at 37 °C in a humidified atmosphere of 5% CO_2_ (Selecta, Barcelona, Spain). The culture medium was changed every other day to avoid nutrient depletion.

### 3.2. Effects of SBA on Intestinal Integrity and Permeability Barrier Function

#### 3.2.1. Measurement of Transepithelial Electrical Resistance (TEER)

IPEC-J2 cells were seeded at 5 × 10^4^ cells/cm^2^ in Millicell membranes with 12 mm diameter size (Millipore, Billerica, MA, USA) and inserted in 24-well transwell plates (Costar, Corning Incorporated, Corning, NY, USA) within 2 days. When the cell monolayer was completely differentiated, cells were treated with 0, 0.5, 1.0, 1.5, 2.0, 2.5, or 3.0 mg/mL SBA, which was extracted and purified by the Animal Production and Product Quality Security Key Laboratory of Jilin Agricultural University (purity and activity were consistent with those of the standard SBA product from Sigma (Aldrich Inc, St. Louis, MO, USA) [48]. TEER was measured after incubating cells for 24, 48, or 72 h with a Millicell-ERS (Millipore, Billerica, MA, USA). Each well was tested 3 times. TEER values were calculated as mean values × Transwell membrane area (0.6 cm^2^).

#### 3.2.2. Enzyme Assay: Determination of Alkaline Phosphatase Activity

Next, we used alkaline phosphatase (AP) assays to analyze the cellular membrane permeability of intestinal epithelial cells indirectly. IPEC-J2 cells were seeded at 5 × 10^4^ cells/cm^2^ in 96-well plates and completely differentiated for 2 days at 37 °C in an atmosphere of 5% CO_2_ and 95% O_2_. Cells were treated with 0, 0.5, 1.0, 1.5, 2.0, 2.5, or 3.0 mg/mL SBA and the treatment time was referred to as 72 h [16]. Culture supernatants were collected, and AP activity was determined using an AP activity assay kit (Nanjing Jiancheng Bioengineering Institute, Nanjing, China) according to the manufacturer’s instructions.

### 3.3. Effects of SBA on Intestinal Epithelial Cell Viability

#### 3.3.1. MTT Assay

MTT assays are generally used as a sensitive method to measure living cell viability and provide evidence for SBA-induced cellular damage.

For MTT assays, IPEC-J2 cells were seeded at 5 × 10^4^ cells/cm^2^ in 96-well plates (Costar, Corning Incorporated, Corning, NY, USA) and completely differentiated for 2 days at 37 °C in an atmosphere of 5% CO_2_ and 95% O_2_. Cells were then treated with 0, 0.5, 1.0, 1.5, 2.0, 2.5, or 3.0 mg/mL SBA for 24, 48, or 72 h. Medium was discarded, and the wells were washed twice with phosphate-buffered saline (PBS). The medium was replaced with 180 μL fresh DMEM/F12 supplemented with 5% FBS and 1% penicillin-streptomycin. Next, 20 μL of MTT solution (Sigma, Aldrich Inc, St. Louis, MO, USA) was added to each well and incubated for 4 h at 37 °C. After incubation, the reaction mixture was carefully removed, and 150 μL dimethyl sulfoxide (DMSO, Solarbio, Shanghai, China) was added to each well and mixed thoroughly for 10 min. The final color was read at 570 nm using a microplate reader (Bio-Rad, Hercules, CA, USA). Data were reported as the mean ± SD from 4 independent experiments.

#### 3.3.2. Cell Morphological Observation

IPEC-J2 cells were seeded at 5 × 10^4^ cells/cm^2^ in 6-well plates and cultured for 24 h at 37 °C in an atmosphere of 5% CO_2_ and 95% O_2_. After treatment with 0, 0.5 or 2.0 mg/mL SBA for 24 h, cell morphology was observed by contrast microscopy.

### 3.4. Effects of SBA on the Distribution and Expression of TJ Proteins

#### 3.4.1. Immunofluorescence

IPEC-J2 cells were grown on glass slides within 6-well plates (Nest, Beijing, China) for 48 h and were then treated with 0 or 0.5 mg/mL SBA for 48 h. After washing with PBS 3 times, the cells were fixed with cold acetone for 30 min at room temperature. Next, the cells were permeabilized with 0.5% Triton X-100 for 3 min, and blocked with 5% bovine serum albumin (BSA) for 30 min at 37 °C. After washing, samples were incubated with rabbit anti-claudin-3 and rabbit anti-occludin antibodies (diluted 1:50 and 1:100, respectively, in 1% BSA; Bioss, Cambridge, MA, USA) for 2 h at 37 °C. Cells were washed 3 times and incubated with FITC-conjugated goat anti-rabbit IgG (CWBIO, Beijing, China) diluted 1:30 for 2 h at room temperature. After washing, images were captured using a laser scanning confocal microscope (Nikon, Tokyo, Japan) and analyzed with NIS-Elements F 3.0 software (Nikon, Tokyo, Japan).

#### 3.4.2. Preparation of Cell Total Protein Extracts

IPEC-J2 cells seeded in cell culture flasks were grown until reaching good confluence and were treated with 0 or 0.5 mg/mL SBA for 48 h for analysis of the expression of TJ proteins. After a wash with PBS, cells within culture flasks were then dissolved in 500 μL cell lysis buffer (20 mM Tris-HCL, pH 8.0, 5 mM EDTA, and 1% Triton X-100) supplemented with protease inhibitors for 30 min on ice. Samples were sonicated 3 times for 20 s each and centrifuged (10000 × *g*, 30 min, 4 °C). Supernatants were collected, and protein concentrations were measured using a BCA kit (Thermo, Waltham, MA, USA).

#### 3.4.3. SDS-PAGE and Western Blotting

Fifteen microliters of 1 mg/mL total protein extracts was separated by sodium dodecyl sulfate polyacrylamide gel electrophoresis (SDS-PAGE) and transferred onto nitrocellulose for 45 min using the semidry transfer film method. The membranes were blocked in 3% nonfat milk overnight at 4 °C. The blots were then washed with modified D-PBS Tween-20 (PBST) and incubated with rabbit anti-claudin-3, rabbit anti-occludin, or rabbit anti-β-actin antibodies (diluted 1:50, 1:100, and 1:1000 in PBS, respectively) for 1.5 h at 37 °C. After washing, they were incubated with goat anti-rabbit IgG HRP-conjugated secondary antibodies (diluted 1:1500, Tianjin Sungene Biotech Co, Ltd, Tianjin, China) for 1.5 h at room temperature. Antibody binding was detected using tabletting and autoradiographic film. Grey levels were determined by Quantity One software (Bio-Rad, Hercules, CA, USA).

### 3.5. Statistical Analysis

Data were analyzed with SPSS software 17.0 using least significant difference (LSD) tests. Differences were considered statistically significant when the *p*-value was less than 0.05.

## 4. Conclusions

We determined the effects of SBA on tight junction protein expression in intestinal epithelial cells in piglets to explain the damaged impact on epithelial permeability and intestinal barrier function. Our data demonstrated that SBA decreased the expression and distribution of occludin and claudin-3, and damaged the permeability of epithelial cells, thereby decreasing the barrier function of intestinal epithelial cells. This study provides an important theoretical basis for understanding the mechanism of the anti-nutritional value of SBA, provides a basis to improve soybean protein utilization efficiency in animals and provides the basic theory for safeguarding human food and animal feeds.

## Figures and Tables

**Table 1:** Pearson correlations between two of the experimental indicators (MTT, AP, and TEER values) after treatment with different concentrations of SBA for 72 h. Pearson correlations were obtained using SPSS 17.0 software.

	Heading	MTT	AP	TEER
MTT	Pearson correlation	1		
	N	28		

AP	Pearson correlation	−0.682 *	1	
	N	28	28	

TEER	Pearson correlation	0.688 *	−0.940 *	1
	N	21	21	21

* indicated significant pearson correlations between the two of the experimental indicators (*p <* 0.01). AP had a significant negative correlation with MTT (*p <* 0.01); TEER had a significant positive correlation with MTT (*p* < 0.01) and a significant negative correlation with AP (*p <* 0.01).
